# Do changes in traditional coronary heart disease risk factors over time explain the association between socio-economic status and coronary heart disease?

**DOI:** 10.1186/1471-2261-11-28

**Published:** 2011-06-03

**Authors:** Peter Franks, Paul C Winters, Daniel J Tancredi, Kevin A Fiscella

**Affiliations:** 1 Center for Healthcare Policy and Research, University of California at Davis, University of California, Davis, 4860 Y Street, Suite 2300, Sacramento, California, 95817, USA; 2 Department of Family Medicine, University of Rochester, 1381 South Ave, Rochester, NY 14620, USA; 3 Center for Healthcare Policy and Research, University of California at Davis, 2103 Stockton Blvd., Suite 2224, Sacramento, California 95817, USA; 4 Department of Family Medicine, University of Rochester, 1381 South Ave, Rochester, NY 14620, USA; 5 Department of Family & Community Medicine, University of California at Davis, 4860 Y Street, Suite 2300, Sacramento, California, 95817, USA; 6 Department of Pediatrics, University of California at Davis, 2103 Stockton Blvd., Suite 2224, Sacramento, California 95817, USA; 7 Departments of Family Medicine and Community & Preventive Medicine, and Department of Oncology, Wilmot Cancer Center, University of Rochester 1381 South Ave, Rochester, NY 14620, USA

**Keywords:** coronary disease, cholesterol, epidemiology, prevention, risk factors

## Abstract

**Background:**

Socioeconomic status (SES) predicts coronary heart disease independently of the traditional risk factors included in the Framingham risk score. However, it is unknown whether *changes *in Framingham risk score variables over time explain the association between SES and coronary heart disease. We examined this question given its relevance to risk assessment in clinical decision making.

**Methods:**

The Atherosclerosis Risk in Communities study data (initiated in 1987 with 10-years follow-up of 15,495 adults aged 45-64 years in four Southern and Mid-Western communities) were used. SES was assessed at baseline, dichotomized as low SES (defined as low education and/or low income) or not. The time dependent variables - smoking, total and high density lipoprotein cholesterol, systolic blood pressure and use of blood pressure lowering medication - were assessed every three years. Ten-year incidence of coronary heart disease was based on EKG and cardiac enzyme criteria, or adjudicated death certificate data. Cox survival analyses examined the contribution of SES to heart disease risk independent of baseline Framingham risk score, without and with further adjustment for the time dependent variables.

**Results:**

Adjusting for baseline Framingham risk score, low SES was associated with an increased coronary heart disease risk (hazard ratio [HR] = 1.53; 95% Confidence Interval [CI], 1.27 to1.85). After further adjustment for the time dependent variables, the SES effect remained significant (HR = 1.44; 95% CI, 1.19 to1.74).

**Conclusion:**

Using Framingham Risk Score alone under estimated the coronary heart disease risk in low SES persons. This bias was not eliminated by subsequent changes in Framingham risk score variables.

## Background

Low socio-economic status (SES) predicts coronary heart disease independent of traditional risk factors included in the Framingham risk score [1-7], particularly in high income countries [8,9]. We previously reported that adding a measure of SES to CHD risk assessment based on Framingham risk scoring also improves model calibration (i.e. corrects under estimation of risk) for low SES persons [1,10]. This finding suggests that consideration of SES in the context of clinical decision making for cholesterol treatment may help address SES disparities in coronary heart disease [11]. Specifically, correction of risk underestimation for low SES persons may allow for better alignment between risk and treatment (e.g. more aggressive cholesterol lowering treatment) for low SES persons, potentially reducing disparities. Importantly, this risk-based approach is intended to augment, rather than supplant, broader approaches to disparities CHD risk [12].

A lingering concern with the above approach is the possibility that the independent association of SES with coronary heart disease is largely explained by changes in Framingham risk score variables over time. For example, low SES persons are less likely to stop smoking [13], and often have less access to care, including less use of medications shown to decrease coronary heart disease risk [14]; thus, SES may act through effects on Framingham risk score variables over time. If such a pathway explained the independent baseline association of SES with subsequent coronary heart disease, it would not be necessary to adjust risk stratification and treatment goals for differences in SES. Periodic updating of Framingham Risk Scoring would be sufficient to account for the effect of SES on CHD risk.

Most prior studies evaluating putative coronary heart disease risk factors (whether behavioral or biomedical) have focused on whether the putative risk factor *measured at baseline *exerts an influence on subsequent coronary heart disease independent of baseline risk factors included in the Framingham risk score. A few studies have examined the impact of *changes in risk factors *on SES-associated cardiovascular risk [15,16]. However, prior studies have not specifically examined whether changes in Framingham risk factors affect the relationship between SES and CHD incidence. We examined data from the Atherosclerosis Risk in Communities (ARIC) study to assess the impact of changes over time in Framingham risk score variables on the relationship between baseline SES and subsequent coronary heart disease.

## Methods

### Sample

This study is based on publicly available ARIC data. It conformed to the Helsinki Declaration and local regulations. It was approved by the University of Rochester and the University of California, Davis Institutional Review Boards. All patients were consented for participation in the original ARIC study from which this secondary data analysis is performed.

ARIC was implemented in four US communities and designed to examine the natural history of atherosclerosis [17]. Field centers randomly selected and recruited a sample of approximately 4,000 individuals aged 45-64 years in Forsyth County, North Carolina (75% urban), Jackson, Mississippi (100% urban), Minneapolis suburbs, Minnesota (100% urban), and Washington county, Maryland (57% urban). In 1987-9, participants were examined, yielding medical and socio-demographic data. Follow-up is ongoing, with examinations every three years. Roughly 75% of eligible respondents participated in the baseline interview and 80% of these participated in the baseline examination [18].

There were 15,732 participants in the study. We excluded the 17% of participants reporting coronary heart disease or equivalents (stroke, peripheral vascular disease, or diabetes) at baseline because current guidelines recommend aggressive treatment for this group (and they are not scored in the Framingham risk scoring system). We also excluded less than 1% of subjects with missing Framingham risk score data elements resulting in a study sample of 12,684. Because of missing SES information (primarily income) 12,139 persons were included in the analyses, with 3315 (27%) in the low SES category (defined below). In terms of missing follow-up data, 5% missed one eligible follow-up examination, and 1.5% missed two. Missing follow-up data were more common among low SES persons (12%) than among high SES persons (4%).

Details regarding data collection and analytic methods for ARIC are published elsewhere [19]. ARIC subjects were asked to fast for 12 hours before their examination visits. For lipid testing, samples were sent to the Central Lipid Laboratory (Houston, Texas). Total cholesterol was determined by enzymatic methods. High density lipoprotein (HDL) cholesterol was measured after dextran-magnesium precipitation [20]. Systolic blood pressure was measured three times, five minutes apart using a random zero sphygmomanometer while the participant was seated. The average of the measures was used for the analysis. Information on smoking status (smoking or not) and anti-hypertensive medication use (or not) were obtained by self-report.

Data regarding coronary heart disease events and risk factors were collected through annual telephone interviews, follow-up examinations every three years, surveys of hospital discharge data, and death certificates from state vital statistics offices [17].

### SES

We used a dichotomous measure of SES (low SES or high SES) using income and education; the measure had been previously validated [1]. We defined persons as low SES if they had < 12 years of schooling and/or had household incomes < $12,000 (corresponding to 50% above the US federal poverty level for income an average U.S. household in 1987) [21]. This simplification of SES was adopted to facilitate its easy incorporation into clinical risk stratification and treatment goals [1-4,11,22,23].

### Framingham Risk Scoring

We used Framingham risk scoring to derive the 10-year risk for a coronary heart disease event or death for men and women as proposed in the National Cholesterol Education Program. Framingham risk scoring uses participant age (10 categories), gender (male/female), total cholesterol (five categories), HDL cholesterol (four categories), smoking status (yes/no), systolic blood pressure (five categories), and use of antihypertensive agents (yes/no) among persons with two or more major coronary heart disease risk factors (smoking, hypertension, low HDL, family history of premature coronary heart disease, and age) to assign points. The precise categories for each variable and the point scoring system that we used have been published elsewhere [24].

### Coronary Heart Disease Events

We assessed the timing (to the day) of the first of any coronary heart disease event up to 10 years following enrollment. Subjects not observed to develop coronary heart disease within 10 years were considered censored (or who died of other causes prior to 10 years). We followed the Atherosclerosis Risk in Communities study criteria for the diagnosis of incident coronary heart disease; diagnosis was based on EKG and cardiac enzyme criteria, or death certificate data and arbitrated by an Atherosclerosis Risk in Communities physician panel [25].

### Time Dependent Variables

In addition to baseline measurement, data on blood pressure, total and HDL cholesterol, smoking status, and use of anti-hypertensive medication were collected during the course of three follow-up examinations conducted every three years.

### Analyses

Analyses used STATA (version 11.1, StataCorp, College Station, TX). We used Cox proportional hazards analyses to assess the influence of baseline and time-varying covariates on the incidence of the initial CHD event during a 10-year follow-up period. Four models were developed. Model 1 examined the effect of SES alone and Model 2 examined the effect of the Framingham risk score alone. Model 3 included both the baseline Framingham risk score and SES. Model 4 added the time dependent variables reflecting the values at each follow-up visit: systolic blood pressure, total and HDL cholesterol, smoking status, and use of anti-hypertensive medication. These time-dependent variables were included as change scores from their respective baseline values.

To better reflect the influence of the Framingham risk score on CHD incidence, the Framingham risk score (on a probability scale) was modeled as its complementary log-log transform (log[-log[1-Framingham risk score]]). Effect measure modification of the SES-CHD association was examined by gender, race (Black vs. White), Framingham risk score and by diastolic blood pressure. None of these additional variables made statistically significant contributions and the results of these analyses are not reported. The proportional hazards assumption of the Cox models were assessed graphically and statistically. No evidence for a substantive departure from proportionality was observed for any of our covariates, although a slight departure was observed with the transformed baseline FRS score toward the end of the 10-year follow-up period. Alternative parameterizations of FRS eliminated the departure from nonproportionality but at the expense of substantially poorer overall model fit and with only slight differences in the coefficients for the other terms in the model. In light of this, we retained the original specification of the baseline FRS score (that used the complementary log-log transformation).

## Results

Table 1 shows the characteristics of the study sample by SES at baseline and the three follow-up examinations. Low SES persons compared with high SES persons were older, and had higher baseline Framingham risk scores, reflecting their higher blood pressures and cholesterols, greater likelihood to smoke and to be on anti-hypertensive medication. There were 456 coronary heart disease events during follow-up; the 10-year CHD incidence was 3.1% in high SES persons and 5.2% in low SES persons. The higher coronary heart disease risk among low SES persons persisted throughout follow-up and changes in risk favored high SES persons throughout.

**Table 1:** Baseline and follow-up characteristics of study sample

	Higher SES		Lower SES	
	**Mean (SD)**	**N**	**Mean (SD)**	**N**

**Baseline**				

Age (years)	53.3 (5.6)	8824	55.2 (5.7)	3315

Male	44.9%	8824	40.7%	3315

Framingham risk score	5.8 (6.0)	8824	7.0 (6.6)	3315

Total cholesterol (mg/dL)	212.3 (40.1)	8824	216.4 (43.7)	3315

HDL cholesterol mg/dL)	52.7 (17.0)	8824	52.9 (17.2)	3315

Systolic blood pressure (mm Hg)	118.0 (16.9)	8824	125.2 (20.0)	3315

Current smoker	22.9%	8824	34.9%	3315

Anti-hypertensive medication	17.8%	8785	28.8%	3300

**3-year follow-up**				

Total cholesterol (mg/dL)	207.8 (37.8)	8451	211.0 (40.3)	2928

HDL cholesterol (mg/dL)	50.5 (16.8)	8424	50.3 (16.8)	2922

Systolic blood pressure (mm Hg)	119.2 (17.5)	8483	125.1 (20.2)	2945

Current smoker	19.8%	8467	29.6%	2932

Anti-hypertensive medication	20.4%	8457	31.9%	2928

**6-year follow-up**				

Total cholesterol (mg/dL)	206.6 (36.4)	7844	208.8 (39.4)	2500

HDL cholesterol (mg/dL)	53.1 (18.3)	7843	52.1 (17.8)	2500

Systolic blood pressure (mm Hg)	122.2 (17.8)	7869	128.7 (20.5)	2515

Current smoker	15.9%	7848	23.5%	2494

Anti-hypertensive medication	25.6%	7834	37.4%	2498

**9-year follow-up**				

Total cholesterol (mg/dL)	200.7 (35.9)	6974	202.1 (38.8)	1990

HDL cholesterol (mg/dL)	50.6 (16.6)	6974	49.4 (16.1)	1990

Systolic blood pressure (mm Hg)	125.3 (18.1)	6994	130.6 (19.5)	2001

Current smoker	13.0%	6969	19.5%	1991

Anti-hy2ertensive medication	30.5%	6965	42.3%	1988

Notes: SES = socio-economic status; SD = standard deviation; HDL = high density lipoprotein.

Table 2 summarizes the results of the Cox proportional hazards models. In the model including baseline Framingham risk score and SES (Model 3), low SES was associated with an increased independent risk for coronary heart disease; the adjusted effects of both were smaller than when SES (Model 1) or Framingham risk score (Model 2) were included alone. When the time dependent risk factors were added to the model (Model 4), the risk associated with SES remained significant.

**Table 2:** Predictors of 10-year coronary heart disease without and with adjustment for time dependent traditional risk factors

	Model 1	Model 2	Model 3	Model 4
	**HR (95% CI)**	**HR (95% CI)**	**HR (95% CI)**	**HR (95% CI)**

Baseline Risk Factors				

Framingham Risk Score		2.29 (2.08, 2.53)	2.26 (2.05, 2.49)	2.25 (2.03, 2.50)

Lower SES	1.79 (1.49, 2.16)		1.53 (1.27, 1.85)	1.44 (1.19, 1.74)

Time Dependent Risk Factors				

Total cholesterol (per 10 mg/dL change)				1.07 (1.03,1.10)

HDL cholesterol (per 10 mg/dL change)				1.02 (0.90,1.14)

Systolic blood pressure (per 10 mm Hg change)				1.12 (1.07,1.17)

Current Smoker				1.58 (1.05,2.39)

Anti-hypertensive medication				0.79 (0.57,1.09)

**Notes: **HR = adjusted hazard ratio; CI = confidence interval; SES = socio-economic status; HDL = high density lipoprotein. Framingham risk score was complementary log log transformed: log (-log (1-Framingham risk score)). Model 1 includes SES only; Model 2 includes Framingham risk score only; Model 3 includes both SES and Framingham risk score; Model 4 adds changes in time dependent risk factors. Time dependent risk factors are changes in value of risk factor from baseline (current-baseline). Blood pressure and lipid change scores were rescaled (by dividing by 10) to produce more interpretable hazard ratios for these measures.

## Discussion

This is the first study to examine whether changes in risk factors over time included in the Framingham risk score could account for the effects of a putative social risk factor for coronary heart disease. Specifically, we assessed the hypothesis that the association of SES with coronary heart disease adjusted for baseline measures of traditional CHD risk factors is explained by changes in those risk factors over time. We found that accounting for these changes explained little of the risk associated with SES.

No studies to our knowledge have examined the effect of changes in FRS on the risk associated with SES on CHD incidence. However, our findings are broadly consistent with other studies examining cardiovascular disease. Stringhini *et al *examined the impact of baseline health behaviors (rather than factors included in Framingham risk scoring) and their changes over time on the association of SES with cardiovascular mortality in British civil servants [16]. They found that baseline health behaviors explained 29% of the effect of SES on subsequent cardiovascular mortality. Notably, however, subsequent health behavior *changes over time *accounted for only 16% of the SES effect on cardio-vascular mortality. Yan *et al *examined the effect of baseline systolic blood pressure, smoking, waist circumference, physical activity, and total cholesterol on coronary artery calcium (CAC), a marker of subclinical atherosclerosis. Consistent with our findings, baseline adjustment had an appreciable effect on the risk associated with SES, but adjustment for *changes *over 15 years had little effect [15].

Our findings reinforce the current United Kingdom recommendations of considering an individual's SES in assessing cardiovascular risk [22,26]. Specifically, our findings show that changes in Framingham risk factors explain little of the social risk for CHD. Thus, ignoring SES in risk stratification and treatment goals may result in undertreatment of low SES persons who are at higher risk for cornary heart disease than their Framingham risk score suggests. SES does not appear to be simply a proxy for poor access and adherence (though those factors are likely also important).

Our study was not designed to address pathways beyond the risk factors included in the Framingham risk score that may explain the higher coronary heart disease incidence among low SES persons. Previous studies suggest that low SES during childhood predicts early coronary heart disease independent of traditional risk factors [5,27]. A growing body of evidence suggests that exposure to social disadvantage and adversity in childhood may result in lasting adaptation to stress, potentially through epigenetic effects [28]. In addition, cumulative effects of social disadvantage across the life course adversely impact cardiovascular health [29]. Such chronic stress appears to exact a physiological toll, likely through multiple, complex pathways involving the hypothalmic-pituitary-adrenal axis, autonomic nervous and immune systems [30]. Thus, SES health effects may represent a proxy measure for life-long "wear and tear." While these and other pathways may be important in explaining how SES exerts its toll on CHD (and directly addressing these pathways may be important), it remains true that clinical decision-making based on Framingham risk scoring alone will under-estimate CHD risk in low SES persons.

Limitations to our findings merit comment. We did not include other biological (such as coronary calcium or C-reactive protein) or behavioral (such as obesity or exercise) risk factors because none are included in Framingham risk scoring currently used in cholesterol risk stratification and treatment guidelines. A prior analysis showed few consistent relationships between a variety of inflamatory markers and social mobility [5].

Participants' reports of smoking, changes in smoking, and anti-hypertensive medication use were not verified. Error in assessment of these risk factors, particularly if associated with SES bias, could result in underestimation of the contribution of these factors. Conversely, repeated measurement of these risk factors and the use of continuous cholesterol and blood pressure measures compared with the single baseline measurement of the dichotomous SES risk factor likely results in a measurement bias favoring the traditional risk factors. We were not able to assess changes in SES during the study period. For example, recent involuntary unemployment is associated with increases in inflammation [31] and higher cardiovascular mortality in some [32-34], but not all [35] studies. Failing to account for these changes in SES would result in misclassification of SES and result in a conservative estimate of the net effect of SES on coronary heart disease.

Missing follow-up data is another potential limitation. While missing follow-up data was relatively uncommon (6% overall), it was more common among low SES persons than among high SES persons. The direction of potential bias is difficult to estimate, depending on whether those with missing data were less or more likely to have changed their level of risk, and whether that change, if any, occurred differntially by SES. However, given the relatively small overall impact of risk factor change on the SES hazard ratio, we consider it unlikely that the potential bias would change our conclusion that there is a robust independent effect of SES on CHD.

In summary, we found that accounting for changes in key traditional coronary heart disease risk factors and anti-hypertensive medication explained little of the independent effect of SES on coronary heart disease risk. Ignoring SES in coronary heart disease risk assessment under-estimates the risk in lower SES persons [1,3], and may, in turn, through relative undertreatment contribute to widening SES disparities in coronary heart disease. These findings provide further support for inclusion of SES into coronary heart disease risk assessment; methods to do so have been presented elsewhere [22,23].

## Conclusion

Using Framingham Risk Score alone under estimated the coronary heart disease risk in low SES persons. This bias was not eliminated by subsequent changes in Framingham risk score variables.

## Competing Interests

The authors declare that they have no competing interests.

## Authors' contributions

PF led the design of this sub-study and writing of the manuscript. PW performed the data analysis. DT provided statistical advice. KF obtained funding for, and directed the parent project. Each of the authors contributed to analysis and interpretation of the data, the manuscript preparation and approved the final version manuscript.

## Pre-publication history

The pre-publication history for this paper can be accessed here:

http://www.biomedcentral.com/1471-2261/11/28/prepub
